# Anaphylaxis to Protamine During a Carotid Endarterectomy

**DOI:** 10.7759/cureus.68289

**Published:** 2024-08-31

**Authors:** Arya Kermanshah, Mariana Rubini Silva Ceschim, Daniel Montes de Oca, Gisele J Wakim

**Affiliations:** 1 Anesthesiology, University of Miami Miller School of Medicine, Miami, USA

**Keywords:** protamine anaphylaxis, unfractionated heparin reversal, allergy and anaphylaxis, peri-operative anaphylaxis, protamine sulfate

## Abstract

Protamine sulfate is commonly used to reverse the anticoagulant effects of unfractionated heparin (UFH) during surgical procedures, but its administration can sometimes trigger severe adverse reactions, including life-threatening anaphylaxis. We present the case of a 77-year-old male undergoing carotid endarterectomy who developed profound hypotension and tachycardia following protamine infusion. Anaphylaxis was confirmed by elevated tryptase levels. This case emphasizes the importance of vigilant monitoring during protamine administration, particularly in high-risk patients, and highlights the need to consider alternative reversal strategies to enhance patient safety.

## Introduction

Protamine is the only readily available and approved agent to reverse the anticoagulation of unfractionated heparin (UFH) and is routinely used after cardiac or vascular procedures. During a carotid endarterectomy (CEA), a bolus of heparin is given before clamping the carotid artery, and reversal with protamine is left to the discretion of the surgeon depending on the bleeding risk. When given, it rapidly binds to UFH and presents a half-life of approximately 10 minutes [1]. Recent meta-analyses explored the benefits and risks of using protamine to reverse heparin after CEA [2]. They found that protamine reduces the need for reoperation due to bleeding without increasing the risk of stroke, heart attack, or death [2]. However, protamine’s effects on hemostasis and hemodynamics require attention. While it is known to neutralize heparin, protamine alone can act as an anticoagulant, inhibiting platelet function and clot strength. Thus, proper dosing is crucial to avoid bleeding risks. The ideal protamine-to-heparin ratio remains uncertain, as does the optimal infusion rate. Additionally, protamine administration can lead to hypotension, which may have benefits but also risks. The inclusion of different closure methods in CEA studies introduces heterogeneity, potentially affecting stroke risk. Given these complexities, protamine should be used judiciously, especially in patients with suspected residual heparin levels, until further research clarifies its risks and optimal usage in CEA [2].

Protamine, a medication utilized to reverse the anticoagulant effects of heparin, is a polycationic low-molecular-weight protein, primarily derived from salmon sperm [1]. Its alkaline nature, predominantly composed of arginine, enables it to effectively neutralize heparin-induced anticoagulation. Protamine, available both in recombinant and original forms, is employed in various clinical scenarios, including cardiopulmonary bypass, dialysis, vascular procedures, and acute ischemic strokes, to mitigate the risk of postoperative bleeding and reverse the effects of heparin. Moreover, protamine has historical significance, initially utilized to prolong the action of insulin preparations [1].

The mechanism of action of protamine involves its interaction with UFH, forming an inactive salt aggregate devoid of anticoagulant properties [1]. Rapid onset and relatively short half-life characterize protamine’s action, necessitating careful dosing to avoid adverse effects such as bleeding. Administration typically occurs intravenously, with a slow infusion recommended to mitigate potential side effects. However, determining the appropriate dose remains contentious, with variations documented in medical literature [1]. Protamine’s efficacy in reversing heparin can be assessed through clotting time measurements or thromboelastogram tests, with ascending aorta infusion emerging as a potentially preferable route [1].

Adverse effects associated with protamine administration encompass anaphylactic responses, pulmonary hypertension, platelet dysfunction, and interference with coagulation factors [1]. Protamine-induced pulmonary hypertension, attributed to thromboxane A2 release, underscores the importance of preventive measures and prompt treatment interventions. Monitoring for adverse reactions, particularly in high-risk patients, alongside access to cardiovascular rescue drugs and resuscitation equipment, is essential. Additionally, understanding protamine’s toxicity, attributed to its positive charge, informs clinical management strategies and underscores the importance of vigilant administration and monitoring protocols [1].

Protamine administration can lead to various adverse effects, notably anaphylactic reactions characterized by systemic hypotension, pulmonary vasoconstriction, allergic responses, and bronchoconstriction, occurring in up to 10.6% of cases [1]. Liver and kidney tissue damage have also been reported. Excess protamine can negatively affect platelet function, interfere with coagulation factors, and promote clot breakdown. Furthermore, it can induce pulmonary hypertension and hypotension, the former attributed to thromboxane A2 release. Management involves treatments such as albuterol, methylprednisolone, antihistamines, vasopressors, and echocardiography. Patients with prior protamine exposure, vasectomy, fish allergies, or protamine-containing insulin-controlled diabetes are at increased risk of adverse reactions [1].

## Case presentation

A 77-year-old male with a history of hypertension, diabetes, cerebrovascular disease, and no known allergies presented with dizziness and difficulty walking. The workup demonstrated severe stenosis of the left internal carotid artery, and the patient was determined to require a left CEA by neurosurgery.

The patient underwent general anesthesia with continuous monitoring of blood pressure (BP), cerebral oximetry, and sensory and motor-evoked potentials. Baseline vitals were BP 168/62, heart rate (HR) 49, and SpO2 100%. Induction was performed with lidocaine, propofol, etomidate, and rocuronium at 11:19 AM. Total IV anesthesia was maintained with propofol and remifentanil. Antibiotics were administered, and the procedure began at 12:20 PM. A low-dose phenylephrine infusion was used to maintain higher mean arterial pressures (MAPs). At 1:15 PM, 5,000 units of heparin were administered at the surgeon’s request, and the procedure proceeded uneventfully.

**Figure 1:**
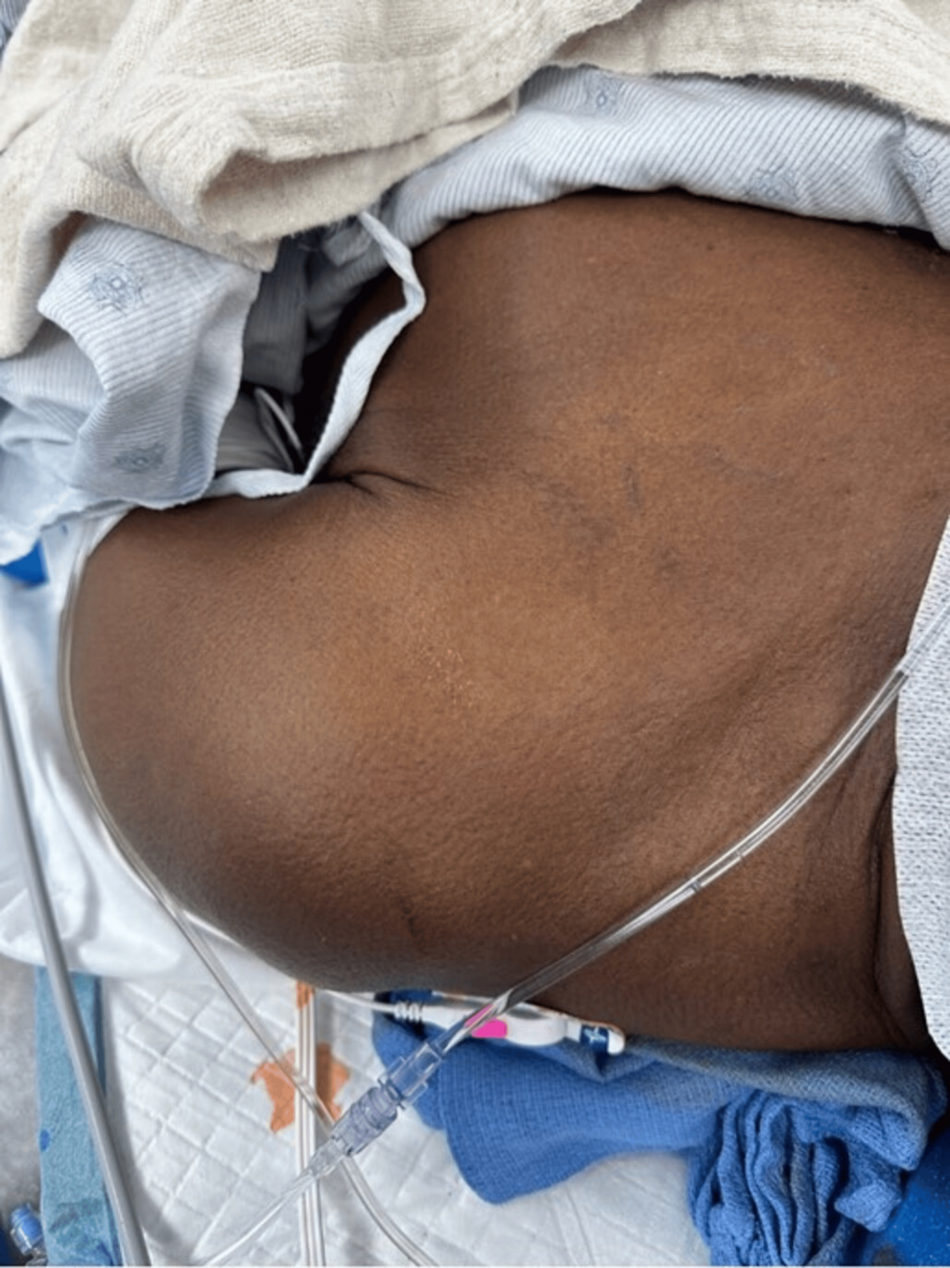
Rash and urticaria observed on the patient’s left shoulder and chest after the drapes were removed

At 1:44 PM, 50 mg of protamine, diluted in 50 mL of NaCl 0.9%, was infused over 10 minutes through a peripheral IV access. At 1:55 PM, the patient developed tachycardia and severe refractory hypotension, with a MAP of 40. At 2:00 PM, additional help was called. Fluid resuscitation was initiated, and increasing doses of IV epinephrine boluses were administered (20-20-60-100 mcg; 200 mcg total), along with vasopressin. A mild transient increase in both plateau and peak inspiratory pressures was noted. An arterial blood gas was obtained, and a tryptase level was sent. At this time, the tubing used for protamine administration was completely removed. At 2:05 PM, the patient was given sodium bicarbonate 50 mEq and hydrocortisone 100 mg, with vitals showing BP 56/44 (MAP 48) and HR 129. The patient’s BP remained labile until approximately 2:20 PM, when he became hemodynamically stable. Surgery concluded at 2:24 PM. When the drapes were removed, hives were noted on the patient’s torso (Figure 1). The patient was extubated at 2:39 PM. In this case, the patient’s tryptase level was 20.6 mcg/L (levels of 11.5 ng/mL or higher may indicate anaphylaxis), and 24 hours later, it decreased to 6.0 mcg/L.

## Discussion

A comprehensive review of both prospective and retrospective studies on protamine administration has found that the incidence of severe anaphylactic reactions ranges from 0.06% to 10.6% [1]. Patients with a history of protamine exposure, vasectomy, fish allergies, and insulin-controlled diabetes are at increased risk [1]. UFH boosts the activity of antithrombin, a natural inhibitor of the clotting cascade, thereby inactivating thrombin and factor Xa, along with other factors. UFH has a short half-life, allowing for rapid reversal by halting its infusion. Protamine sulfate is the licensed reversal agent for UFH, acting by blocking its interaction with antithrombin. However, protamine sulfate itself can act as an anticoagulant, so dosing is calculated based on the units of UFH received in the last two hours, with 1 mg of protamine sulfate neutralizing approximately 80-100 units of UFH [3]. Vasodilation is common and may cause mild transient hypotension. One study defines the vasodilatory effects of protamine on systemic circulation and evaluates its dependency on dose and endothelium. They used vascular rings from rabbit abdominal aortas and found that protamine induces endothelium-dependent vasodilation, regardless of the presence of heparin. These findings imply a need for caution in high-risk patients to prevent potential cardiovascular collapse induced by protamine, possibly by considering alternative anticoagulation neutralization methods or premedication guidelines [4].

Life-threatening anaphylactic reactions are non-dose-dependent immediate hypersensitivity reactions. They may occur within five to 20 minutes of administration and are usually IgE- or IgG-mediated. The antigen-antibody response to protamine sulfate results in a type I anaphylactic reaction [5]. Signs range from cutaneous to pulmonary or cardiovascular responses. Immediate treatment includes stopping protamine and administration of epinephrine. Blood should be collected for tryptase determination. Tryptase levels in serum serve as a critical marker for allergic reactions, particularly anaphylaxis. A rapid increase in tryptase indicates mast cell degranulation, signaling the severity of the reaction. This measurement aids in distinguishing anaphylaxis from other emergencies, reducing unnecessary investigations. Moreover, it prompts the identification of the triggering agent and cross-reacting substances, enhancing risk management [6]. In this case, the patient’s tryptase was 20.6 mcg/L (levels of 11.5 ng/mL or higher may indicate anaphylaxis), and 24 hours later 6.0 mcg/L. IgE levels were also significantly increased. Measures to prevent/mitigate reactions include a test dose and a slow infusion rate. After discharge, patients who had a severe reaction should be referred to an allergist.

While several options exist for anticoagulation, including direct oral anticoagulants and thrombin inhibitors, protamine remains the approved reversal agent for heparin, with few alternatives being investigated. Despite initially being designed as an antidote for apixaban and rivaroxaban, in vitro studies demonstrate that andexanet can effectively reverse heparin’s anticoagulant effects in a dose-dependent manner, making it a potential alternative for protamine [7].

In diabetic patients with a history of protamine-containing insulin usage, it is important to consider alternative methods to protamine administration when reversing heparinization. Proposed alternatives encompass autoreversal of lower-dose heparinization, employing heparin-bound heart bypass pump circuits to obviate protamine reversal, chemically reversing heparinization with hexadimethrine, or employing investigational agents such as heparinase or platelet factor 4 [8]. Platelet factor 4 and heparinase are currently under investigation and challenging to procure. Hexadimethrine (polybrene), previously utilized for heparin reversal post-cardiac surgery, has been supplanted by protamine primarily due to its potential nephrotoxicity. If unable to implement any of the mentioned alternatives, consider prophylactic use of antihistamines and corticosteroids before administering protamine [8]. The efficacy of this approach lacks support from controlled, prospective studies. Consequently, there is apprehension that premedication may not suffice to prevent IgE-mediated anaphylaxis from protamine, given the historical failure of medication pretreatment in reliably reducing such risks with other agents like chymopapain and penicillin [8].

## Conclusions

Protamine, a crucial agent for reversing heparin’s anticoagulant effects, presents significant clinical considerations due to its potential adverse effects, notably anaphylactic reactions. This review highlights protamine’s mechanism of action, indications, and adverse effects, including anaphylaxis, pulmonary hypertension, and platelet dysfunction. Importantly, it emphasizes the need for vigilant monitoring, prompt recognition, and appropriate management of adverse reactions, particularly in high-risk patients. Furthermore, the discussion underscores the value of alternative approaches to protamine administration and the ongoing research into alternative reversal agents. The case study provided illustrates a clear temporal and causal relationship between protamine administration and anaphylaxis, reinforcing the importance of careful consideration and management of protamine use in clinical practice. Overall, this information is valuable for the medical community in enhancing patient safety and optimizing clinical outcomes during procedures involving protamine administration.
